# No association between head injury with loss of consciousness and Alzheimer disease pathology—Findings from the University of Manchester Longitudinal Study of Cognition in Normal Healthy Old Age

**DOI:** 10.1002/gps.5129

**Published:** 2019-05-10

**Authors:** Andrew C. Robinson, Yvonne S. Davidson, Michael A. Horan, Maggie Cairns, Neil Pendleton, David M.A. Mann

**Affiliations:** ^1^ Faculty of Biology, Medicine and Health, School of Biological Sciences, Division of Neuroscience and Experimental Psychology University of Manchester, Salford Royal Hospital Salford UK; ^2^ Department of Geriatric medicine, Bolton NHS Foundation Trust Royal Bolton Hospital Bolton UK

**Keywords:** head injury, Alzheimer disease, neuropathology

## Abstract

**Objectives:**

Head injury with loss of consciousness (HI‐LOC) is a common occurrence. Some studies have linked such injuries with an increased risk of Alzheimer disease (AD). However, recent large clinicopathologic studies have failed to find a clear relationship between HI‐LOC and the pathological changes associated with AD. The present study aims to further investigate the relationship between HI‐LOC and AD pathology in the elderly.

**Methods/Design:**

History of HI‐LOC in participants in the University of Manchester Longitudinal Study of Cognition in Normal Healthy Old Age was ascertained. The donated brains of 110 of these individuals were assessed for AD pathology using consensus guidelines. Analyses aimed to elucidate relationships between HI‐LOC and AD pathology.

**Results:**

No associations were found between incidence of HI‐LOC and regional AD pathology or any of the three established measures of the neuropathology associated with AD: CERAD score, Thal phase, or Braak stage.

**Conclusions:**

Single incidences of HI‐LOC may not be sufficient to cause the pathology associated with late‐stage AD. Other routes of damage, such as diffuse axonal injury or Lewy body pathology, may play a greater role in causing cognitive impairment associated with head injury.

**Key points**

- Single incidences of head injury with loss of consciousness may not be sufficient to cause the pathology associated with late‐stage Alzheimer disease.
- It is possible that other pathologies are involved in causing the cognitive impairment associated with head injury such as the pathology associated with Parkinson disease.
- Further study is needed to elucidate the relationships between head injury and Alzheimer disease pathology

## 1 INTRODUCTION

Major or sustained trauma to the head, such as that seen in *Dementia pugilistica*, is well known to cause cognitive impairment and dementia. Head injury on the whole has been implicated by the majority of studies^1^ in the development of Alzheimer disease (AD). Previous human post‐mortem studies have found links between the neuropathology of AD and chronic traumatic encephalopathy (CTE), which is common in sports where concussive injury may occur.^2^ Similarly, it has been shown that a single, moderate to severe traumatic brain injury (TBI) may increase both amyloid‐beta (Aβ) and tau deposition^3^, ^4^ and after fatal head injury, accumulation of the pathological form of Aβ, Aβ_1‐42_, can be found.^5^, ^6^

Although there seems to be strong evidence of increased Aβ production after acute TBI,^4^, ^7^, ^8^ it is not clear whether this results in increased Aβ plaque formation^9^ or whether this Aβ remains in a prefibrillar state.^10^ Indeed, Aβ plaques associated with TBI are formed rapidly after injury and are thought to be similar to the “diffuse” plaques found in the elderly and early in AD rather than the “neuritic” plaques found later in the disease course.^11^ Similarly, acute TBI also appears to increase tau levels in CSF and tau hyperphosphorylation, but this does not seem to immediately lead to the classic neurofibrillary tangles of AD.^12^ However, it has been shown that those surviving severe TBI are more likely to exhibit tau pathology at the age of 60 years of age when compared with age‐matched controls.^13^

Recently, it has been suggested^14^ that large, robust clinicopathological and biomarker studies,^15^, ^16^ which avoid the usual limitations of self‐reported TBI in already cognitively impaired individuals, have failed to confirm the relationship of TBI to the development of AD dementia or AD pathology. The present study aims to further investigate the relationship of TBI and AD pathology in the elderly.

## 2 MATERIALS AND METHODS

### 2.1 Participants and study design

Full details concerning recruitment of participants and study design of the University of Manchester Longitudinal Study of Cognition in Normal Healthy Old Age has been previously described.^17^

For the present study, one specific questionnaire, the Cornell Medical Index (CMI) was used to gain insight into each individual's medical history and lifestyle.

The CMI questionnaire was administered to all participants up to five times between 1994 and 2007. The CMI included detailed checklists of physical and mental problems for each individual to complete. The area of interest for the present study was whether they had ever received head injury with loss of consciousness (HI‐LOC). The question was posed in the CMI questionnaire as “Were you ever knocked unconscious?” A positive indication on any of the CMI tests was recorded. CMI scores have been validated against clinical examinations in a number of previous studies.^18^

Cognitive status at death was ascertained using a combination of last score on the modified Telephone Interview for Cognitive Status (TICSm) before death, patient notes obtained via participants' general practitioner, and cause of death as recorded on the death certificate. A TICSm score of below 21 was used to define cognitive impairment.^19^ Individuals were assigned either normal for age (at death) or cognitively impaired.

### 2.2 Pathological methods

A total of 312 individuals consented to donate their brain after death, and so far, 110 of these brains have been collected. A full description of the pathological methods and the overall neuropathological profile of the cohort has been previously described.^20^

Paraffin sections (6 μm) were immunostained for Aβ (Cambridge Bioscience, clone 4G8, 1:3000) and tau proteins phosphorylated at Ser202 and Thr205 (P‐tau) (Innogenetics, clone AT8, 1:750). For antigen retrieval, sections were either immersed in 70% formic acid for 20 minutes (for Aβ) or microwaved in 0.1M citrate buffer, pH 6.0 (for tau) prior to incubation with primary antibody.

Alongside regional, semiquantitative pathology scores (Table S1), a CERAD score, Thal phase, and Braak stage were assigned to assess AD pathology.^21^, ^22^, ^23^

### 2.3 Statistical analyses

Chi‐squared test was used to analyse whether there were differences in sex, age group at death (younger than 90 y vs older than 90 y) and cognitive impairment between those who has suffered HI‐LOC and those who had not.

To assess the impact of HI‐LOC on AD pathology, a positive indication of HI‐LOC was correlated with regional pathology, CERAD score, Thal phase, and Braak stage (Spearman rank correlation).

A *P* value of <.05 was considered significant.

## 3 RESULTS

Demographics and characteristics of the University of Manchester Longitudinal Study of Cognition in Normal Healthy Old Age cohort are shown in Table 1. Of the 110 individuals in the study, 30 (27%) had reported a prior HI‐LOC. Females outnumbered males by approximately 2:1. The median age at death for the cohort was 89 years old, and there was approximately equal numbers of individuals in the assigned age groups of younger than 90 years old and 90 years old or older. Most participants were considered cognitively normal at death, but there was a sizable subset (39%) of individuals who were found to be cognitively impaired at death. No significant differences were found between those who had experienced HI‐LOC and those who had not for sex, age group at death, or cognitive status.

**Table 1:** Characteristics of the University of Manchester Longitudinal Study of Cognition in Normal Healthy Old Age cohort stratified by HI‐LOC status

Characteristic	No HI‐LOC (n = 80)		HI‐LOC (n = 30)		Total (n = 110)		P Value
	n	%	n	%	n	%	
Sex							
Male	24	30	11	37	35	32	.504
Female	56	70	19	63	75	68	
Age at death							
Younger than 90 y	44	55	18	60	62	56	.638
90 y+	36	45	12	40	48	44	
Median age at death (range)	89 (31)		88 (29)		89 (32)		
Cognitive status							
No cognitive impairment	48	60	19	63	67	61	.750
Cognitively impaired	32	40	11	37	43	39	

Abbreviation: HI‐LOC, head injury with loss of consciousness.

**Figure 1:**
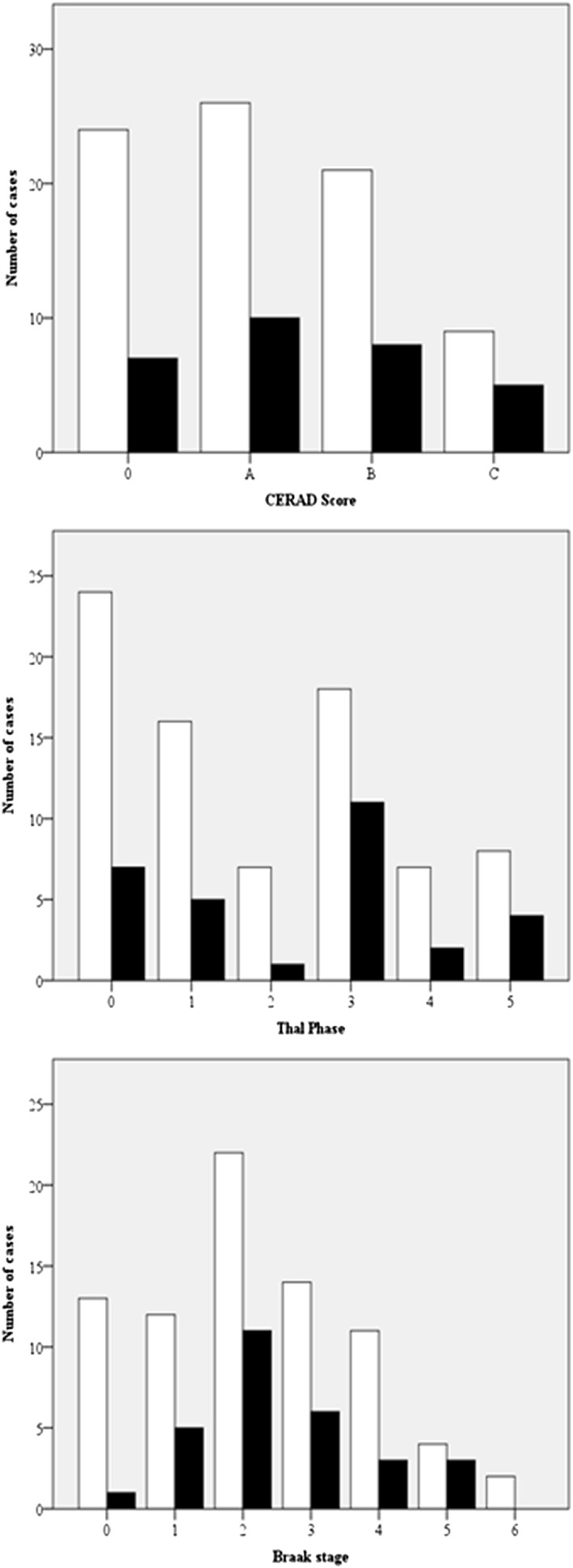
Frequency of Alzheimer disease (AD) pathology in the University of Manchester Longitudinal Study of Cognition in Normal Healthy Old Age cohort stratified by head injury with loss of consciousness (HI‐LOC) status. Those with HI‐LOC are shown in black and those without are shown in white

The relationship between HI‐LOC and occurrence of AD pathology is shown in Figure 1 and Table 2.

**Table 2:** Spearman rank correlation coefficients and associated P values for associations between HI‐LOC and various measures of regional AD pathology

		Amyloid‐beta										PHF‐tau							
		F	O	P	T	Am	H	CS	Cb	CERAD	Thal	F	O	P	T	Am	H	Cb	Braak
HI‐LOC	*r*_*s*_	.113	.162	.118	.002	.099	.076	.185	.067	.077	.095	.059	.183	.030	.095	.089	.103	.024	.078
	*P*	.238	.090	.219	.986	.316	.434	.055	.487	0.422	.322	.540	.056	.758	.323	.367	.290	.801	.425

Abbreviations: F, frontal; O, occipital; P, parietal; T, temporal; Am, amygdala; H, hippocampus; CS, corpus striatum; Cb, cerebellum; HI‐LOC, head injury with loss of consciousness.

No significant correlations were found between head injury groups and any of the semiquantitative scores for regional AD pathology.

CERAD stage A, which corresponds to uncertain evidence of AD in the over 75‐year‐old age group, was most common in both individuals with HI‐LOC and those with no history of HI‐LOC. No significant correlation was found between head injury groups and CERAD score (*r*_*s*_ = .077, *P* = .422).

In those without history of HI‐LOC, Thal phase 0 was most common although a significant proportion (23%) exhibited Thal phase 3. In those with HI‐LOC, Thal phase 3 was most common. However, there was a lack of correlation between head injury groups and Thal phase (*r*_*s*_ = .095, *P* = .322).

Braak stage II was found to be most common in both those with and without HI‐LOC. Again, no significant correlation was found between head injury groups and Braak stage (*r*_*s*_ = .078, *P* = .425).

## 4 DISCUSSION

In this study, analysing 110 individuals from the University of Manchester Longitudinal Study of Cognition in Normal Healthy Old Age cohort who had consented to brain donation, we found no association between incidence of cognitive impairment and HI‐LOC nor did we find any correlation between HI‐LOC and regional AD pathology or any of the three established measures of the neuropathology associated with AD: CERAD score, Thal phase, or Braak stage. Although the present study is limited by the self‐reporting nature of HI‐LOC and lack of knowledge of exact date and nature of head injury, our findings mirror those found in the two larger, well‐powered clinicopathological studies^14^ and suggest that HI‐LOC may not be a risk factor for increased severity of AD‐associated neuropathological lesions.

It may be the case that single incidents of HI‐LOC are not enough of an insult to cause the pathology (and therefore clinical presentation) associated with late‐stage AD. A single blow might favour deposition of Aβ through enhanced APP production and catabolism following TBI,^24^ but this does not seem to exacerbate tau pathology. Frequency of injury may be as important as severity when considering neuropathological outcomes.

It is worthy of note that those with HI‐LOC were most commonly found to be at Thal phase 3 (although there was no significant correlation found). Thal stage assesses total Aβ deposition, and Thal phase 3 can be considered a “crossover” phase as both those with and those without cognitive impairment have been found to exhibit this feature.^22^

Although no associations were found between HI‐LOC and AD pathology in the present study, it is clear from previous studies that TBI has some impact on risk of clinical dementia. It could be postulated that the underlying mechanism of cognitive impairment found in such cases may not be AD pathology. Previous studies have shown other possible routes of damage including diffuse axonal injury within the white matter of TBI patients^25^ and pathology associated with Parkinson disease.^15^ Therefore, TBI is not completely innocuous, and further study is required to address the long‐term consequences of this phenomenon.

## 5 CONCLUSION

Rare or single occurrences of HI‐LOC may not be sufficient to cause late‐stage AD pathology. It is possible that other routes of damage, such as diffuse axonal injury or Lewy body pathology, may play a greater role in causing cognitive impairment associated with head injury.

## CONFLICT OF INTEREST

None declared.

## AUTHOR CONTRIBUTIONS

David Mann and Neil Pendleton devised and designed the study and helped with writing the paper. D.M. finalised neuropathological diagnosis. N.P. and Maggie Cairns finalised clinical cognitive impairment diagnosis. Andrew Robinson helped to devise and design the study, performed immunohistochemistry, microscopic assessments, genetic analysis, and data/statistical analysis, and wrote the paper. Yvonne Davidson helped with immunochemistry. Michael Horan helped to finalise clinical cognitive impairment diagnosis, provided clinical data and assisted with preparation of the manuscript.

## RESEARCH ETHICS COMMITTEE APPROVAL

The study was approved by Manchester Brain Bank Management Committee (REC reference 09/H0906/52 + 5). Under conditions agreed with the Research Ethics Committee, The Manchester Brain Bank can supply tissue or data to researchers, without requirement for researchers to apply individually to the REC for approval.

## DATA AVAILABILITY STATEMENT

The data that support the findings of this study are available from the corresponding author upon reasonable request.

## Supporting information

Table S1. Semi‐quantitative regional scores for amyloid‐beta and PHF‐tau and associated CERAD score, Thal phase and Braak stage
