# Supplementary material for: No association between head injury with loss of consciousness and Alzheimer disease pathology—Findings from the University of Manchester Longitudinal Study of Cognition in Normal Healthy Old Age
Source: Int J Geriatr Psychiatry. 2019 May 10;34(8):1262–6. doi: 10.1002/gps.5129 (PMC6767119; doi:10.1002/gps.5129)
Supplement: Supplementary file 1 — Table S1. Semi‐quantitative regional scores for amyloid‐beta and PHF‐tau and associated CERAD score, Thal phase and Braak stage [file GPS-34-1262-s001.docx]

| Case ID | HI-LOC | **Amyloid-beta** | | | | | | | | | | **PHF-tau** | | | | | | | |
| --- | --- | --- | --- | --- | --- | --- | --- | --- | --- | --- | --- | --- | --- | --- | --- | --- | --- | --- | --- |
|  |  | F | O | P | T | Am | H | CS | Cb | CERAD | THAL | F | O | P | T | Am | H | Cb | BRAAK |
| 05//05 | Yes | 3 | 2 | 3 | 2 | 2 | 2 | 2 | 0 | B | 3 | 0 | 0 | 0 | 1 | 1 | 3 | 0 | III |
| 06//03 | No | 3 | 2 | 3 | 3 | 2 | 1 | 3 | 1 | A | 5 | 1 | 0 | 2 | 3 | 2 | 2 | 0 | IV |
| 06//08 | No | 2 | 3 | 2 | 2 | 3 | 2 | 2 | 0 | B | 3 | 2 | 2 | 2 | 3 | 3 | 2 | 0 | IV |
| 06//11 | No | 3 | 2 | 3 | 2 | 3 | 1 | 2 | 0 | C | 4 | 1 | 1 | 3 | 3 | 2 | 1 | 0 | V |
| 06//12 | No | 1 | 1 | 1 | 1 | 0 | 0 | 0 | 0 | A | 0 | 0 | 0 | 0 | 0.5 | 0.5 | 0 | 0 | I |
| 06//19 | No | 2 | 2 | 3 | 3 | 2 | 1 | 2 | 0 | C | 3 | 0.5 | 1 | 1 | 2 | 2 | 1 | 0 | IV |
| 07//06 | No | 3 | 2 | 3 | 3 | 2 | 2 | 3 | 0 | C | 4 | 2 | 0.5 | 2 | 3 | 3 | 2 | 0 | IV |
| 07//07 | No | 0 | 0 | 1 | 0 | 0 | 0 | 0 | 0 | 0 | 0 | 0.5 | 0 | 0 | 1 | 0.5 | 1 | 0 | II |
| 07//10 | Yes | 3 | 2 | 2 | 2 | 1 | 1 | 3 | 0 | C | 3 | 0.5 | 1 | 0.5 | 2 | 1 | 1 | 0 | III |
| 07//13 | No | 0 | 1 | 0 | 0 | n/a | n/a | 0.5 | 0 | 0 | 0 | 0 | 0 | 0 | 0.5 | n/a | n/a | 0 | 0 |
| 08//02 | No | 0 | 0 | 0 | 0 | 0 | 0 | 0 | 0 | 0 | 0 | 0 | 0 | 0 | 1 | 1 | 1 | 0 | III |
| 08//04 | No | 2 | 3 | 1 | 2 | 2 | 1 | 2 | 0 | A | 4 | 0.5 | 0 | 0 | 0.5 | 0.5 | 1 | 0 | II |
| 08//28 | No | 2 | 2 | 2 | 2 | 1 | 0 | 2 | 0 | A | 3 | 0 | 0 | 0 | 0.5 | 0.5 | 0 | 0 | 0 |
| 08//29 | Yes | 2 | 3 | 2 | 2 | 3 | 1 | 2 | 1 | A | 5 | 0.5 | 3 | 0.5 | 2 | 3 | 2 | 0 | II |
| 09//05 | Yes | 0 | 0 | 0 | 0 | 0 | 0 | 0 | 0 | 0 | 0 | 0 | 0 | 0 | 2 | 0.5 | 1 | 0 | II |
| 09//06 | Yes | 3 | 3 | 3 | 2 | 2 | 1 | 3 | 2 | C | 5 | 3 | 3 | 3 | 3 | 3 | 2 | 0 | V |
| 09//07 | Yes | 3 | 2 | 3 | 2 | 1 | 1 | 3 | 0 | B | 4 | 2 | 0.5 | 3 | 3 | 3 | 3 | 0 | IV |
| 09//11 | No | 0 | 0 | 0 | 2 | 1 | 0 | 1 | 0 | 0 | 1 | 3 | 0 | 0 | 1 | 1 | 1 | 1 | n/a* |
| 09//15 | No | 3 | 2 | 2 | 3 | 2 | 1 | 2 | 0 | C | 4 | 3 | 0.5 | 3 | 3 | 2 | 2 | 0 | IV |
| 09//21 | Yes | 2 | 2 | 2 | 2 | 3 | 2 | 1 | 0 | B | 3 | 3 | 0.5 | 0.5 | 2 | 2 | 2 | 0 | III |
| 09//22 | No | 3 | 3 | 2 | 2 | 3 | 1 | 3 | 0 | B | 3 | 2 | 3 | 1 | 3 | 3 | 2 | 0 | IV |
| 09//24 | No | 0 | 0 | 0 | 0 | 0 | n/a | 0 | 0 | 0 | 0 | 0 | 0 | 0 | 0.5 | 0 | n/a | 0 | 0 |
| 09//26 | Yes | 0 | 0 | 0 | 0 | 0 | 0 | 0 | 0 | 0 | 0 | 0 | 0 | 0 | 0 | 1 | 1 | 0 | I |
| 09//30 | No | 3 | 2 | 2 | 2 | 3 | 1 | 3 | 0 | A | 3 | 0 | 0 | 0 | 2 | 1 | 1 | 0 | II |
| 09//31 | No | 0 | 0 | 0.5 | 0 | 0 | 0 | 0 | 0 | 0 | 0 | 0 | 0 | 0 | 0 | 0.5 | 1 | 0 | I |
| 10//07 | Yes | 3 | 2 | 3 | 2 | 2 | 2 | 3 | 1 | C | 5 | 2 | 2 | 1 | 3 | 2 | 3 | 0 | V |
| 10//08 | No | 2 | 2 | 3 | 2 | 2 | 2 | 2 | 1 | B | 5 | 2 | 3 | 3 | 3 | 2 | 2 | 0 | III |
| 10//16 | Yes | 3 | 2 | 2 | 2 | 2 | 1 | 2 | 0 | B | 3 | 1 | 1 | 2 | 3 | 2 | 2 | 0 | III |
| 10//40 | Yes | 3 | 2 | 3 | 2 | 3 | 1 | 2 | 0 | C | 3 | 0 | 0.5 | 0 | 2 | 1 | 1 | 0 | III |
| 10//41 | No | 1 | 1 | 2 | 2 | 0 | 0 | 0.5 | 0 | 0 | 1 | 0 | 0 | 0 | 0.5 | 0.5 | 0 | 0 | 0 |
| 11//06 | Yes | 1 | 2 | 1 | 1 | 0.5 | 0 | 1 | 0 | A | 1 | 0 | 0 | 0 | 0.5 | 2 | 1 | 0 | II |
| 11//07 | No | 0 | 0 | 0 | 0 | 0 | 0 | 0 | 0 | 0 | 0 | 0 | 0 | 0 | 0.5 | 0.5 | 0 | 0 | 0 |
| 11//15 | No | 1 | 0 | 0.5 | 2 | 2 | 0 | 0 | 0 | 0 | 0 | 0 | 0 | 0 | 2 | 0.5 | 1 | 0 | I |
| 11//20 | No | 3 | 2 | 2 | 2 | 2 | 1 | 1 | 0 | B | 1 | 0 | 0 | 0 | 2 | 2 | 2 | 0 | II |
| 11//22 | No | 0 | 0 | 0 | 0 | 0 | 0 | 0 | 0 | 0 | 0 | 0 | 0 | 0 | 0 | 0.5 | 0 | 0 | 0 |
| 11//25 | No | 0 | 0 | 0 | 0 | 0 | 0 | 0 | 0 | 0 | 0 | 0 | 0 | 0.5 | 2 | 2 | 1 | 0 | II |
| 11//27 | No | 1 | 0.5 | 1 | 0.5 | 1 | 0 | 0.5 | 0 | A | 1 | 0 | 0 | 0 | 1 | 2 | 2 | 0 | II |
| 11//29 | No | 0 | 0.5 | 0 | 0 | 0 | 0 | 0 | 0 | 0 | 0 | 0 | 0 | 0.5 | 0.5 | 1 | 1 | 0 | II |
| 11//30 | No | 0 | 0.5 | 1 | 0 | 0 | 0 | 0 | 0 | 0 | 0 | 0 | 0 | 0 | 1 | 1 | 1 | 0 | II |
| 12//09 | No | 1 | 1 | 0.5 | 2 | 2 | 0 | 1 | 0 | A | 1 | 0 | 0 | 0 | 2 | 0.5 | 1 | 0 | I |
| 12//12 | Yes | 3 | 2 | 3 | 3 | 3 | 1 | 3 | 2 | B | 5 | 1 | 2 | 0.5 | 2 | 2 | 2 | 0 | II |
| 12//23 | No | 0 | 0 | 0 | 0 | 0 | 0 | 0 | 0 | 0 | 0 | 0 | 0 | 0 | 0.5 | 0.5 | 1 | 0 | I |
| 12//28 | Yes | 3 | 2 | 3 | 3 | 3 | 2 | 3 | 0 | A | 3 | 0 | 0 | 0.5 | 0.5 | 3 | 1 | 0 | II |
| 12//33 | Yes | 2 | 3 | 2 | 2 | 3 | 1 | 2 | 0 | B | 4 | 3 | 2 | 2 | 3 | 3 | 2 | 0 | IV |
| 12//34 | No | 1 | 2 | 1 | 2 | 1 | 0 | 0.5 | 0 | B | 1 | 2 | 0.5 | 2 | 1 | 2 | 2 | 0 | III |
| 12//35 | No | 3 | 1 | 3 | 2 | n/a | 1 | 3 | 0 | A | 3 | 0 | 0 | 0 | 1 | n/a | 1 | 0 | I |
| 13//10 | No | 3 | 2 | 3 | 2 | 2 | 1 | 2 | 0 | C | 3 | 3 | 3 | 3 | 3 | 3 | 2 | 0 | V |
| 13//11 | Yes | 3 | 2 | 2 | 2 | 2 | 1 | 2 | 0 | C | 3 | 3 | 0.5 | 3 | 3 | 3 | 2 | 0 | V |
| 13//12 | No | 3 | 1 | 2 | 2 | 2 | 1 | 2 | 0 | A | 1 | 1 | 0.5 | 0.5 | 2 | 2 | 2 | 0 | III |
| 13//16 | No | 3 | 3 | 3 | 3 | n/a | 1 | 3 | 1 | B | 5 | 1 | 2 | 2 | 3 | n/a | 3 | 0 | III |
| 13//17 | No | 3 | 2 | 3 | 2 | 2 | 1 | 1 | 0.5 | B | 3 | 1 | 1 | 1 | 3 | 3 | 3 | 0 | IV |
| 13//21 | No | 0 | 0 | 0 | 0 | 0 | 0 | 0 | 0 | 0 | 0 | 0 | 0 | 0 | 0 | 0.5 | 1 | 0 | III |
| 13//22 | Yes | 0 | 0 | 0 | 0 | 0 | 0 | 0 | 0 | 0 | 0 | 0 | 0 | 0 | 0 | 1 | 1 | 0 | II |
| 13//23 | No | 1 | 1 | 2 | 0 | 1 | 0 | 1 | 0 | A | 0 | 0 | 0 | 0.5 | 1 | 2 | 1 | 0 | II |
| 13//31 | Yes | 0 | 0.5 | 1 | 0 | n/a | 0 | 0 | 0 | 0 | 0 | 0.5 | 0 | 0 | 2 | n/a | 3 | 0 | II |
| 13//32 | No | 2 | 1 | 2 | 2 | 2 | 0 | 0.5 | 0 | A | 1 | 0 | 0 | 0.5 | 0.5 | 1 | 0 | 0 | 0 |
| 13//35 | No | 0 | 0 | 0 | 0 | 0 | 0 | 0 | 0 | 0 | 0 | 0 | 0 | 0 | 0.5 | 0.5 | 0 | 0 | II |
| 13//36 | Yes | 2 | 3 | 2 | 2 | 3 | 1 | 2 | 0 | A | 3 | 0.5 | 0.5 | 0 | 2 | 3 | 2 | 0 | II |
| 13//43 | Yes | 0 | 0 | 0 | 0 | 0 | 0 | n/a | n/a | 0 | 0 | 1 | 0 | 0 | 2 | 2 | 1 | n/a | II |
| 14//01 | No | 3 | 1 | 2 | 2 | n/a | 1 | 2 | 0 | B | 4 | 2 | 0.5 | 2 | 3 | n/a | 2 | 0 | IV |
| 14//04 | No | 2 | 1 | 2 | 2 | 2 | 0 | 0 | 0 | A | 1 | 0 | 0 | 0 | 0 | 1 | 0 | 0 | 0 |
| 14//06 | No | 2 | 1 | 2 | 2 | 2 | 1 | 2 | 0 | B | 1 | 1 | 0.5 | 1 | 3 | 3 | 3 | 0 | II |
| 14//11 | No | 0 | 0 | 0 | 0 | 0 | 0 | 0 | 0 | 0 | 0 | 0 | 0 | 0 | 0 | 1 | 0 | 0 | I |
| 14//14 | Yes | 3 | 2 | 3 | 3 | 3 | 1 | 3 | 0 | B | 3 | 1 | 1 | 0.5 | 1 | 3 | 2 | 0 | III |
| 14//15 | No | 3 | 2 | 2 | 2 | 2 | 1 | 2 | 0 | B | 3 | 1 | 0.5 | 1 | 2 | 2 | 1 | 0 | II |
| 14//16 | No | 1 | 1 | 1 | 2 | 3 | 0 | n/a | 0 | A | 1 | 0 | 0 | 0 | 0 | 0 | 0 | 0 | 0 |
| 14//20 | No | 0.5 | 0.5 | 0.5 | 1 | 0.5 | 0 | 0 | 0 | A | 1 | 0.5 | 0 | 0 | 0 | 1 | 1 | 0 | 0 |
| 14//26 | No | 1 | 0.5 | 1 | 1 | 0 | 0 | 0.5 | 0 | A | 1 | 0.5 | 0 | 1 | 0.5 | 3 | 2 | 0 | II |
| 14//29 | No | 2 | 1 | 2 | 2 | 1 | 0 | 1 | 0 | B | 1 | 0.5 | 0 | 0.5 | 1 | 1 | 1 | 0 | III |
| 14//40 | Yes | 2 | 1 | 2 | 1 | 1 | 0 | 2 | 0 | A | 1 | 2 | 0.5 | 1 | 2 | 1 | 2 | 1 | n/a* |
| 14//41 | Yes | 2 | 2 | 3 | 2 | 2 | 0 | 3 | 0 | A | 3 | 0.5 | 0.5 | 1 | 1 | 1 | 1 | 0 | I |
| 14//42 | Yes | 3 | 2 | 3 | 2 | 2 | 1 | 3 | 0.5 | B | 3 | 0.5 | 1 | 1 | 3 | 3 | 2 | 0 | IV |
| 14//46 | No | 0 | 0 | 0 | 0 | 0 | 0 | 0 | 0 | 0 | 0 | 0 | 0 | 0 | 0.5 | 1 | 0 | 0 | 0 |
| 15//01 | Yes | 0.5 | 1 | 1 | 1 | 1 | 0 | 0.5 | 0 | A | 1 | 0 | 0 | 0 | 0.5 | 1 | 0 | 0 | I |
| 15//05 | No | 2 | 0.5 | 1 | 1 | 2 | 0 | 1 | 0 | A | 1 | 0 | 0 | 0 | 0 | 1 | 1 | 0 | I |
| 15//11 | No | 3 | 3 | 3 | 3 | 2 | 1 | 3 | 1 | C | 5 | 2 | 3 | 2 | 3 | 3 | 2 | 0 | VI |
| 15//14 | No | 2 | 2 | 2 | 2 | 2 | 0 | 2 | 0 | B | 3 | 1 | 1 | 1 | 3 | 3 | 1 | 0 | IV |
| 15//15 | No | 0 | 0 | 0 | 0 | 0 | 0 | 0 | 0 | 0 | 0 | 0 | 0 | 0 | 0.5 | 2 | 1 | 0 | II |
| 15//16 | No | 3 | 3 | 3 | 3 | 3 | 2 | 3 | 1 | C | 5 | 3 | 3 | 3 | 3 | 3 | 3 | 2 | VI |
| 15//19 | No | 2 | 1 | 2 | 1 | 2 | 1 | 1 | 0 | B | 2 | 1 | 0 | 1 | 1 | 3 | 3 | 0 | III |
| 15//26 | No | 2 | 2 | 1 | 2 | 1 | 0 | 2 | 0 | A | 3 | 0 | 0 | 0 | 0 | 0.5 | 0 | 0 | 0 |
| 15//28 | No | 0 | 0 | 0 | 0 | 0 | 0 | 0 | 0 | 0 | 0 | 0 | 0 | 0 | 1 | 2 | 1 | 0 | II |
| 15//30 | No | 1 | 1 | 1 | 2 | 2 | 0 | 1 | 0 | A | 2 | 0.5 | 0 | 0 | 1 | 3 | 2 | 0 | II |
| 15//31 | No | 2 | 0.5 | 2 | 2 | 2 | 0 | 2 | 0 | A | 3 | 0 | 0 | 0 | 0.5 | 0.5 | 0 | 0 | I |
| 15//42 | No | 3 | 3 | 3 | 3 | 3 | 1 | 3 | 2 | B | 5 | 0.5 | 2 | 1 | 2 | 3 | 3 | 0 | III |
| 15//44 | Yes | 3 | 2 | 2 | 2 | 1 | 0 | 2 | 0 | A | 1 | 2 | 0.5 | 2 | 3 | 3 | 3 | 0 | 0 |
| 15//47 | No | 2 | 2 | 1 | 2 | 0.5 | 0 | 0 | 0 | 0 | 0 | 1 | 0 | 0 | 2 | 3 | 3 | 0 | II |
| 16//01 | No | 3 | 3 | 3 | 3 | 3 | 1 | 3 | 0.5 | B | 4 | 1 | 0 | 2 | 2 | 3 | 3 | 0 | III |
| 16//03 | No | 1 | 1 | 3 | 2 | 1 | 1 | 3 | 0 | C | 3 | 3 | 1 | 3 | 3 | 3 | 3 | 0 | V |
| 16//12 | No | 0 | 0 | 0 | 0 | 0 | 0 | 0 | 0 | 0 | 0 | 0 | 0 | 0 | 0 | 3 | 3 | 0 | II |
| 16//13 | No | 3 | 3 | 3 | 2 | 3 | 1 | 3 | 1 | C | 5 | 3 | 1 | 3 | 3 | 3 | 3 | 0 | V |
| 16//17 | No | 3 | 2 | 3 | 3 | 2 | 1 | 2 | 0 | A | 3 | 0.5 | 0 | 0 | 1 | 3 | 3 | 0 | II |
| 16//30 | No | 3 | 1 | 2 | 2 | 2 | 2 | 2 | 0 | A | 3 | 0.5 | 0 | 0 | 1 | 3 | 3 | 0 | II |
| 16//37 | No | 2 | 1 | 2 | 2 | 2 | 1 | 1 | 0 | B | 2 | 3 | 0 | 3 | 2 | 3 | 3 | 0 | III |
| 16//41 | No | 3 | 1 | 1 | 2 | 1 | 0 | 0 | 0 | 0 | 0 | 0 | 0 | 0 | 0 | 2 | 1 | 0 | I |
| 16//43 | No | 3 | 2 | 3 | 3 | 3 | 2 | 3 | 0 | A | 1 | 1 | 1 | 1 | 3 | 3 | 3 | 0 | III |
| 16//45 | No | 3 | 3 | 3 | 3 | 1 | 2 | 3 | 1 | A | 1 | 2 | 1 | 1 | 1 | 3 | 3 | 0 | III |
| 17//04 | Yes | 0 | 0 | 0 | 0 | 0 | 0 | 0 | 0 | 0 | 0 | 0 | 0 | 0 | 0 | 2 | 2 | 0 | I |
| 17//08 | No | 2 | 2 | 2 | 2 | 2 | 2 | 0 | 0 | 0 | 0 | 0 | 0 | 0 | 0 | 3 | 3 | 0 | II |
| 17//09 | Yes | 0 | 1 | 0.5 | 0 | 1 | 0 | 0 | 0 | 0 | 0 | 0 | 0 | 0 | 0 | 1 | 1 | 0 | I |
| 17//18 | No | 3 | 2 | 3 | 2 | 2 | 2 | 3 | 0 | A | 0 | 1 | 2 | 2 | 3 | 3 | 3 | 0 | n/a* |
| 17//22 | Yes | 3 | 2 | 2 | 3 | 2 | 2 | 2 | 0 | 0 | 0 | 0 | 0 | 1 | 1 | 3 | 3 | 0 | II |
| 17//29 | No | 2 | 1 | 1 | 2 | 2 | 1 | 1 | 0 | 0 | 0 | 1 | 0 | 1 | 2 | 2 | 2 | 0 | II |
| 17//32 | No | 3 | 1 | 2 | 3 | 3 | 3 | 3 | 0 | A | 0 | 2 | 1 | 2 | 3 | 3 | 3 | 0 | IV |
| 17//34 | No | 0 | 0 | 0 | 0 | 0 | 0 | 0 | 0 | 0 | 0 | 0 | 0 | 0 | 0 | 1 | 1 | 0 | I |
| 17//38 | Yes | 2 | 0 | 1 | 2 | 0 | 0 | 1 | 0 | 0 | 0 | 0 | 0 | 0 | 1 | 2 | 2 | 0 | II |
| 18//03 | No | 0 | 0 | 0 | 0 | 0 | 0 | 0 | 0 | 0 | 0 | 0 | 0 | 0 | 0 | 1 | 1 | 0 | 0 |
| 18//09 | No | 2 | 2 | 2 | 2 | 2 | 1 | 1 | 0 | A | 0 | 3 | 1 | 2 | 3 | 3 | 3 | 0 | IV |
| 18//11 | No | 1 | 1 | 1 | 1 | 0 | 0 | 0 | 0 | 0 | 0 | 0 | 0 | 0 | 1 | 2 | 1 | 0 | I |
| 18//18 | No | 2 | 1 | 2 | 2 | 2 | 2 | 1 | 0 | 0 | 0 | 0.5 | 0 | 1 | 3 | 3 | 3 | 0 | III |

**Supplementary data 1** – Semi-quantitative regional scores for amyloid-beta and PHF-tau and associated CERAD score, Thal phase and Braak stage.

0 = no pathology, 0.5 = rare pathology, 1 = mild pathology, 2 = moderate pathology and 3 = severe pathology.

* = Braak staging not available due to presence of confounding tauopathy.
